# Association of bacterial vaginosis with periodontitis in a cross-sectional American nationwide survey

**DOI:** 10.1038/s41598-020-79496-4

**Published:** 2021-01-12

**Authors:** Cláudia Escalda, João Botelho, José João Mendes, Vanessa Machado

**Affiliations:** 1 Evidence-Based Hub Egas Moniz, Clinical Research Unit (CRU), Centro de Investigação Interdisciplinar Egas Moniz (CiiEM), Egas Moniz - Cooperativa de Ensino Superior, CRL, Almada, Portugal; 2 Periodontology Department, Egas Moniz Dental Clinic, Clinical Research Unit (CRU), Egas Moniz Interdisciplinary Research Center (EMIRC), IUEM, Egas Moniz University, Campus Universitário, Quinta da Granja, Monte de Caparica, Caparica, 2829 - 511 Almada, Portugal

**Keywords:** Biomarkers, Diseases, Health care

## Abstract

To explore the association between bacterial vaginosis (BV) and periodontitis (PD) and to determine whether PD and BV might be linked with systemic serum alterations. We used the National Health and Nutrition Examination Survey 2001–2004, with women aged 18–49 years old and diagnosed with or without BV according to Nugent’s method. PD was defined according to the 2012 case definition. We compared serum counts according to the presence of PD and the presence of BV. Multivariable regression was used to explore and identify relevant variables towards the presence of BV. 961 women fulfilled the inclusion criteria. In women with BV, PD was associated with higher inflammation, characterized by increased white blood cells (*p* = 0.006) and lymphocyte (*p* = 0.009) counts. Predictive models presented a statistically significant association between PD and BV [Odds Ratio (OD) = 1.69, 95% Confidence Interval (CI): 1.09–2.61 for periodontitis; OD = 2.37, 95% CI: 1.30–4.29 for severe PD]. Fully adjusted models for age, smoking, body mass index, diabetes mellitus and number of systemic conditions reinforced this association [OD = 1.71, 95% CI: 1.06–2.76 for PD; OD = 2.21, 95% CI: 1.15–4.25 for severe PD]. An association between BV and PD is conceivable. PD was associated with higher systemic markers of inflammation in women with BV. Our data is novel and could serve as a foundation to guide future studies in the confirmation of this association and the underlying mechanisms.

## Introduction

Bacterial vaginosis (BV) is a common vaginal condition with unknown aetiology which represents a relevant shift in the vaginal microbiota. BV is characterized by a thin gray/white and malodorous “fishy” discharge, with high vaginal pH (> 4.5), and vaginal epithelial cells heavily coated with bacteria^1–4^. The abnormal vaginal discharge is the result of an imbalanced vaginal microbiota with increase of anaerobic microorganisms (e.g., *Gardnerella vaginalis, Prevotella Intermedia, Peptostreptococcus, and Bacteriodes spp*)^1,2,5^. Also, BV is associated with reproductive and obstetric sequelae, increasing women’s risk of pelvic inflammatory disease, spontaneous abortion, preterm delivery, low birth weight, and postpartum endometritis^2,5–9^. Susceptibility to BV is dependent on the host immune response and is multifactorial^1,2,10–12^. Genetics, environmental and physiologic stressors, hormonal variations based on the reproductive status and use of hormonal contraceptives are relevant factors towards BV^1,10^.

Periodontitis (PD) is a chronic multifactorial inflammatory condition characterized by inflamed gums and bone destruction caused by a dysbiotic plaque^13–15^ It is preceded by gum inflammation (gingivitis) and an uncontrolled inflammatory response from the innate and adaptive immune system^16^. PD has also been associated with several chronic and systemic diseases^17–23^. A recent review discussed the available evidence and possible mechanisms between female infertility related conditions as a modifiable risk factor towards PD^24,25^. Particularly, women with BV were associated with a significant diversification of salivary microbiota and higher counts of *Prevotella intermedia* (a PD-related bacteria) in the supragingival microbiota compared with women without BV^26^. However, the mechanism through which the oral microbiota suffers dysbiosis in the presence of BV is not determined^26^.

Data from the Longitudinal Study of Vaginal Flora reported a significant association between PD and BV^27^, and women with BV were also found to have higher risk of gingivitis^28^. Comprehensively, bacterial communities from the vagina and the periodontium have higher counts of *Fusobacteria nucleatum* and *Prevotella intermedia*^27,29–31^. Also, the presence of these bacteria may trigger infections in primed women^27,32^, and may have unpleasant consequences during pregnancy, such as preterm birth and low birth weight^2,5–9^. Further, some studies also support the notion of haematogenous spread or oral-genital direct transfer^27,29,33^, however, the relationship between these two pathologies remains poorly understood.

For this reason, we aimed to investigate the likelihood of an association between BV and PD in a representative American cohort of adult women. Secondly, we explored whether this association might lead to systemic alterations measured via blood samples, through complete blood count (white, red and platelet lineages) and C-reactive protein (CRP) levels.

## Results

### Baseline characteristics

From a total of 21,161 participants in National Health and Nutrition Examination Survey (NHANES) 2001–2002 and 2003–2004, we excluded 10,301 (48.7%) males, 7395 women under 18 years or over 50 years old (34.9%), 301 pregnant women (1.4%) and 38 women who did not perform the pregnancy test or had an invalid result (0.2%). Overall, from 3126 included women, a final sample of 961 completed both BV and periodontal clinical examinations. Table 1 displays the characteristics of the included women participants.

**Table 1:** Participants characteristics.

	Non-BV (n = 510)	BV (n = 451)	*p* value^a^
Age, mean (SD) (years)	32.4 (10.1)	32.0 (10.3)	0.556
**Race/ethnicity, n (%)**			
Mexican American	122 (51.1)	117 (49.0)	**0.000**
Non-Hispanics White	258 (63.1)	151 (36.9)	
Non-Hispanic Black	148 (63.0)	87 (37.0)	
Other Hispanic	26 (51.0)	25 (49.0)	
Other race	10 (50.0)	10 (50.0)	
**Education**			
< High school	126 (43.8)	162 (56.3)	**0.000**
High school	129 (54.0)	110 (46.0)	
> High school	255 (58.8)	179 (41.2)	
Active smokers, n (%)	95 (9.9)	115 (12.0)	**0.007**
BMI, mean (SD) (kg/m^2^)	27.2 (7.2)	28.2 (8.3)	**0.017**
**Marital status**			
Single	163 (47.8)	178 (52.2)	**0.002**
Married/living with a partner	299 (58.7)	210 (41.3)	
Divorced/separated/widowed	48 (47.5)	53 (52.5)	
**Medical conditions, n (%)**			
Diabetes mellitus	17 (1.8)	19 (2.0)	0.325
Other medical conditions	0.5 (0.8)	0.6 (0.9)	0.528
Periodontal health, n (%)	472 (49.1)	397 (41.3)	**0.013**
**Periodontitis staging, n (%)**			
Mild (stage I)	21 (51.2)	20 (48.8)	
Moderate (stage II)	15 (39.5)	23 (60.5)	
Severe (stage III)	2 (15.4)	11 (84.6)	
PISA	11.8 (26.4)	13.9 (27.8)	**0.012**
PESA	93.5 (232.0)	87.0 (260.4)	0.543

Significant correlations are identified in bold (*p* < 0.05).

BV, patients with bacterial vaginosis; n, number; SD, standard deviations; Non-BV, patients without bacterial vaginosis; PESA, periodontal epithelial surface area; PISA, periodontal inflamed surface area; <, higher than; >, less than.

Values are given as mean (standard error) or % (standard error).

^a^Chi-square test for categorical variables, Mann–Whitney test for continuous variables, *p* < 0.05.

The prevalence of PD in the overall sample was 9.6% (54% in patients with BV and 38% in non-BV women), although women with BV had higher prevalence (*p* = 0.013). Women with BV had a higher percentage of active smokers (12.0%), higher levels of body mass index (BMI) (28.2 kg/m^2^) and presenting with higher education levels (56.3%) when compared to participants with no clinical signs of BV. Indeed, the number of medical conditions recorded was similar in both groups. Mean ± Standard deviation (SD) of periodontal inflamed surface area (PISA) and periodontal epithelial surface area (PESA) were 11.8 ± 26.4 and 93.5 ± 232.0 in patients without BV versus 13.9 ± 27.8 and 87.0 ± 260.4 for those with BV, respectively (*p* = 0.012 for PISA and *p* = 0.543 for PESA).

### Hematologic counts

The complete blood count was used to compare the blood counts of the PD group with the periodontally healthy women, according to the BV diagnosis. Globally, women with PD presented higher counts of white blood cells (WBC) (*p* = 0.019) and lymphocytes (*p* = 0.021), and lower counts of monocyte (*p* = 0.050). Specifically in women without BV, PD was associated with lower counts of monocytes (*p* = 0.013). Women diagnosed with both BV and PD presented increased counts of WBC (*p* = 0.006), lymphocytes (*p* = 0.009) and CRP (*p* = 0.045) (Table 2).

**Table 2:** Hematologic counts according to bacterial vaginosis and periodontal state.

Variables, mean (SD)	BV (n = 510)			Non-BV (n = 451)			Global (n = 961)		
	Non-PD (n = 472)	PD (n = 38)	*p* value^a^	Non-PD (n = 397)	PD (n = 54)	*p* value^a^	Non-PD (n = 869)	PD (n = 92)	*p* value^a^
WBC count (10^9^/L)	7.2 (2.6)	8.2 (2.7)	**0.006**	7.0 (2.5)	7.1 (2.6)	0.857	7.1 (2.5)	7.8 (2.7)	**0.019**
Monocyte (%)	7.0 (2.6)	6.8 (2.3)	0.578	7.0 (2.3)	6.2 (2.6)	**0.013**	7.0 (2.5)	6.6 (2.5)	**0.050**
Monocyte (10^9^/L)	0.5 (0.2)	0.5 (0.2)	0.387	0.5 (0.2)	0.5 (0.2)	0.192	0.5 (0.2)	0.5 (0.2)	0.993
Segmented neutrophils (%)	55.8 (15.5)	55.7 (15.2)	0.896	57.0 (13.7)	57.0 (17.2)	0.469	56.4 (14.6)	56.2 (16.0)	0.660
Segmented neutrophils (10^9^/L)	4.2 (1.8)	4.4 (2.1)	0.086	4.2 (1.8)	4.4 (2.1)	0.646	4.3 (1.9)	4.6 (2.2)	0.094
Eosinophils (%)	2.4 (2.5)	2.3 (1.8)	0.810	2.3 (2.1)	1.7 (1.1)	0.058	2.4 (2.3)	2.1 (1.6)	0.178
Eosinophils (10^9^/L)	0.2 (0.2)	0.1 (0.1)	0.735	0.2 (0.2)	0.1 (0.1)	0.266	0.2 (0.2)	0.2 (0.1)	0.662
Basophils (%)	0.6 (0.5)	0.6 (0.4)	0.988	0.6 (0.4)	0.5 (0.5)	0.080	0.7 (0.4)	0.3 (0.3)	0.281
Basophils (10^9^/L)	0.0 (0.1)	0.0 (0.1)	0.075	0.0 (0.1)	0.0 (0.1)	0.339	0.0 (0.1)	0.0 (0.1)	0.361
Lymphocyte (%)	29.6 (10.6)	30.6 (10.7)	0.474	29.4 (9.2)	28.8 (10.9)	0.506	29.5 (9.9)	29.9 (10.7)	0.850
Lymphocyte (10^9^/L)	2.1 (0.8)	2.5 (0.8)	**0.009**	2.1 (0.7)	2.1 (0.7)	0.914	2.1 (0.8)	2.3 (0.8)	**0.021**
RBC count (million cells/µL)	4.3 (1.0)	4.4 (0.7)	0.211	4.3 (0.9)	4.3 (1.2)	0.398	4.3 (1.0)	4.4 (0.9)	0.613
Hemoglobin (g/dL)	12.6 (3.1)	13.0 (2.4)	0.395	12.9 (2.8)	12.8 (3.6)	0.501	12.8 (2.9)	12.9 (2.9)	0.890
Hematocrit (%)	37.3 (8.9)	38.7 (6.8)	0.301	38.0 (8.2)	38.2 (10.5)	0.642	37.7 (8.5)	38.4 (8.4)	0.668
MCV (fL)	83.6 (19.4)	85.5 (14.4)	0.488	85.0 (17.7)	83.0 (22.0)	0.240	84.3 (18.5)	84.5 (17.7)	0.791
MCH (pg)	28.3 (6.7)	28.8 (5.2)	0.585	28.9 (6.2)	28.0 (7.6)	0.179	28.6 (6.4)	28.5 (6.2)	0.617
MCHC (g/dL)	32.3 (7.1)	33.0 (4.8)	0.416	32.7 (6.5)	31.6 (8.1)	0.100	32.5 (6.8)	32.5 (6.3)	0.657
RDW (%)	12.3 (3.0)	12.8 (2.4)	0.144	12.2 (2.7)	12.4 (3.8)	0.575	12.2 (2.8)	12.6 (3.0)	0.420
Platelet count (10^9^/L)	278.9 (91.4)	297.4 (82.7)	0.183	281.6 (90.7)	292.4 (106.9)	0.672	280.3 (90.9)	295.4 (92.6)	0.197
MPV (fL)	8.0 (1.9)	8.3 (1.6)	0.134	8.0 (1.8)	7.6 (2.1)	0.075	8.0 (1.9)	8.0 (1.8)	0.903
CRP (mg/dL)	0.4 (1.1)	0.7 (1.0)	**0.045**	0.4 (1.0)	0.4 (0.6)	0.939	0.4 (1.0)	0.6 (0.9)	0.093

Significant correlations are identified in bold (*p* < 0.05).

BV, patients with bacterial vaginosis; CRP, C-reactive protein; Non-BV, patients without bacterial vaginosis; P(−), no periodontitis, P(+), periodontitis; WBC, white blood cells; RBC, red blood cells; MCV, mean cell volume; MCH, mean cell hemoglobin; MCHC, mean cell hemoglobin concentration; RCD, red cell distribution; MPV, mean platelet volume.

^a^Mann–Whitney test for continuous variables, *p* < 0.05.

**Figure 1:**
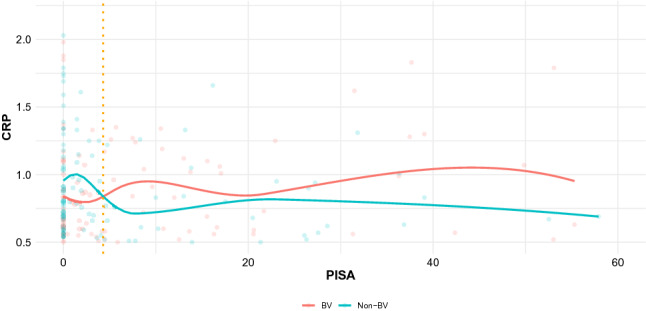
Comparison of C-reactive protein (CRP) (ng/mL) according to Periodontal Inflamed Surface Area (PISA) for women with and without BV.

Then, we graphically explored the behaviour of CRP levels of women with and without BV according to the levels of PISA (Fig. 1). In BV women, CRP levels tend to increase with the increase of PISA levels. Up to 4.4 levels of PISA, women with BV presented lower levels of CRP than non-BV counterparts.

### Predictive models of periodontitis on bacterial vaginosis patients

In the crude model (model 1), the association of PD with BV was statistically significant [odds ratio (OR) = 1.69, 95% confidence interval (CI): 1.09–2.61 for PD; OR = 2.37, 95% CI: 1.30–4.29 for severe PD] (Table 3). Also, the fully adjusted models with age, smoking habits, BMI, diabetes mellitus and number of systemic conditions confirmed a significant association (OR = 1.71, 95% CI: 1.06–2.76 for PD; OR = 2.21, 95% CI: 1.15–4.25 for severe PD) (Table 3).

**Table 3:** Odds ratios (OR) and correspondent 95% confidence intervals (CI) towards bacterial vaginosis, according to the periodontal status, calculated within binary logistic regression analyses for different adjustment levels.

	Periodontitis OR (95% CI)	Severe periodontitis OR (95% CI)
Model 1	1.69 (1.09–2.61)*	2.37 (1.30–4.29)**
Model 2	1.76 (1.13–2.74)*	2.52 (1.37–4.63)**
Model 3	1.66 (1.04–2.65)*	2.29 (1.21–4.33)*
Model 4	1.67 (1.04–2.69)*	2.14 (1.12–4.09)*
Model 5	1.68 (1.04–2.70)*	2.16 (1.13–4.12)*
Model 6	1.71 (1.06–2.76)*	2.21 (1.15–4.25)*

Model 1, unadjusted model; Model 2, includes adjustment for age; Model 3, includes adjustment for age and smoking habits; Model 4, includes adjustment for age, smoking habits and BMI; Model 5, includes adjustment for age, smoking habits, BMI and diabetes mellitus; Model 6, includes adjustment for age, smoking habits, BMI, diabetes mellitus and number of systemic diseases (excluding diabetes). Statistically significant: *p < 0.05; **p < 0.01; ***p < 0.001.

## Discussion

We aimed to investigate whether BV and PD were associated in a representative nationwide sample of women Americans. Our results support our initial hypothesis of a possible link between BV and PD. Women with BV had higher prevalence of PD, levels of periodontal inflammation, percentage of active smokers and average BMI. Also, women with BV and PD presented significantly more systemic inflammation than those with healthy periodontium.

Thus so far, this study is the first to explore the likelihood of a systemic association via inflammatory hallmarks between BV and PD. Our findings emphasize that when both BV and PD co-exist, a significant systemic inflammatory load is detected (measured as CRP) and may be dependent on the periodontal inflammation (measured as PISA). Further, BV patients with PD present higher counts of white and lymphocyte cells than BV patients without PD.

Considering the leukocyte data in this study, our results are consistent with the available evidence, where an increase in WBC is expected from an indirect impact of PD^34,35^. Still, the counts of neutrophils were not a significant parameter in this analysis, despite their strong involvement in the pathogenesis of periodontal disease as they contribute to tissue destruction by releasing toxic products, such as enzymes and cytokines^36–38^. BV is commonly defined by the absence of neutrophils in vaginal swabs observed by flow cytometry^4,39,40^, and therefore an impact on circulating neutrophil counts might be unlikely to exist. However, since the periodontal examination was partially employed, this may have influenced these contradictory results.

The reported prevalence of BV in this American population is consistent with the report by Coudray and Madhivanan^41^ which shows a prevalence of 29.2%. Others, Javed et al.^42^ and Peebles et al.^43^ both found BV to be more prevalent in Non-Hispanic Black women with 51.4% and 33.2%, respectively. However, our results demonstrate higher rates in Non-Hispanic White women. Also, our result contrasts with the prevalence of BV in NHANES 2001–2004^44^. A possible explanation for those differences may be due to the data mining when evaluating patients with both BV and PD examinations, that resulted in the exclusion of a substantial percentage of the overall sample (44.4%).

Our results are in accordance with previous literature stating there is a higher number of women who were diagnosed with BV and had smoking habits. In this sense, smoking habits has a significant effect on innate immunity, and it has been shown to have varying effects on markers of cervicovaginal immunity, namely with higher interleukin (IL)-10 levels in cervicovaginal secretions^10,45–47^. It is also a major modifiable risk factor for PD^48–50^ and it is hypothesized that smoking may lead to an increase in the prevalence of periodontal pathogens as well as delays the neutrophils recruitment into periodontal tissues, and thus compromise the immune response^16,51–56^. Further, our results also show that women with BV had higher BMI compared with women without BV, and this result is in contrast with Loken et al.^57^ result, however this can be explained due to the distinct types of populations and environmental factors.

These results might contribute to increase awareness of obstetricians/gynecologists for the higher risk of women with BV towards the presence of PD and the elevated inflamed burden that these patients may be exposed to.

Further, the coexistence of BV and PD in pregnant women may have severe consequences. Several lines of evidence indicate that infectious processes are associated with preterm delivery, namely overt or subclinical intrauterine infection, lower genital tract infection and distant infections, such as periodontal infection^58^. On the one hand, periodontal bacteria and/or their pathogenic subproducts may spread into the bloodstream and reach the foeto-placental unit triggering an inflammatory response^59^. On the other hand, inflammatory cytokines and mediators (such as CRP) due to periodontal inflammation also play a key role, perpetuating this inflammatory response^59^. Lastly, high levels of maternal CRP in early pregnancy are associated with preterm birth^60–62^, and PD might be seen as a contributing element for this unpleasant outcome.

Undoubtedly, BV may cause genital mucosal inflammation^3^, though evidence on local cervicovaginal cytokine levels in BV is still debatable^3,63–65^. Nevertheless, the consequences of maintained elevated systemic CRP levels in women with BV are unknown given the lack of clinical evidence. On the other hand, PD is a recognized factor towards persistent inflammatory states on a plethora of other chronic diseases, for instance in diabetes, cardiovascular diseases or rheumatoid arthritis^17,66–68^. Further, the treatment of gum disease is effective in alleviating this inflammatory burden^69–71^. Therefore, future studies are warranted to unveil whether treating PD in women with BV might mitigate the inflammatory load observed in this study and the added value of doing so.

The present study is an epidemiological representative study of a segment of the American population composed of women in fertile age. A big strength of this study is the size of the data set used, comprising two NHANES waves. BV was assessed using Nugent’s score which is widely regarded as the gold standard for the diagnosis of BV in research and clinical studies^72–75^, and we employed the most up-to-date periodontal classification^76^. Also, these results raise awareness on the interplay between oral health and women genital tract health and stress the fact that there is a need for more research to conclude a possible relationship between both diseases as well as to understand their linking mechanisms.

There are, however, some limitations to mention. Due to its novelty, we are not able to compare our results. Also, NHANES waves stated in this paper used a partial index to assess periodontal status which diminishes the sensitivity and specificity^77,78^, and this may limit the validity of the established association duo to a possible underestimation of PD. Hence future studies shall implement full-mouth periodontal charts to contribute to more consistent results. Furthermore, this is a cohort study that precluded the inference of causality or temporal relationship between the analyzed variables.

### Conclusions

Women with BV have higher risk of presenting PD, and therefore an association between BV and PD is possible. Also, women with BV and PD present significantly higher systemic markers of inflammation than those without PD. Future studies are warranted to ascertain further this association as well the mechanisms upon this interplay.

## Materials and methods

### Study design and participants

We conducted a secondary analysis on data from the NHANES, a stratified multistage national representative survey with civilian noninstitutionalized population in fifty states of the United States of America (USA) and the District of Columbia. All specific details about the sampling, design, medical records and periodontal data-collections are available at www.cdc.gov/nchs/nhanes.htm. Both NHANES 2001–2002 and 2003–2004 were reviewed and approved by the Centers for Disease Control (CDC) and Prevention National Center for Health Statistics Research (NCHS) Ethics Review Board, and all included participants provided written informed consent.

For the purpose of this study, NHANES 2001–2002 and NHANES 2003–2004 data was retrieved with the following inclusion criteria: women between the ages of 18 and 49 years; non-pregnant; who received periodontal examination and the assessment to BV. This secondary study was performed following the STrengthening the Reporting of OBservational studies in Epidemiology (STROBE) guidelines^79^.

### Bacterial vaginosis assessment

Self-vaginal swabs were collected and then the swab was onto a glass slide to air dry^80,81^. The smears were processed at Magee-Women’s Hospital (Pittsburgh, Pennsylvania), and the BV score was calculated according to Nugent’s method^82^. In this sense, scores between 0 and 3 were considered normal vaginal microbiota, scores of 7 or higher were considered positive for BV, whereas scores between 4 and 6 were considered intermediate. For analysis, the outcome was defined as BV confirmed (positive) or not (negative or intermediate).

### Periodontal examination

Half-mouth periodontal examination was conducted on randomly assigned quadrants, one upper and one lower, by calibrated examiners as described elsewhere^83,84^. Probing pocket depth (PPD), clinical attachment loss (CAL) and bleeding on probing (BOP) measurements were performed at three sites per tooth (mesio-buccal, mid-buccal and disto-buccal). The diagnosis and staging of PD was carried out according to the CDC and Prevention-American Academy of Periodontology consensus for epidemiologic studies recommendation^85^. As such, mild PD was defined as ≥ 2 interproximal sites with CAL ≥ 3 mm and ≥ 2 interproximal sites with PPD ≥ 4 mm (not on the same tooth) or 1 site with PPD ≥ 5 mm. Moderate PD was defined as ≥ 2 interproximal sites with CAL ≥ 4 mm (not on the same tooth) or ≥ 2 interproximal sites with PPD ≥ 5 mm, also not on the same tooth. Severe PD was defined as the presence of ≥ 2 interproximal sites with CAL ≥ 6 mm (not on the same tooth) and ≥ 1 interproximal site with PPD ≥ 5 mm. The sum of mild, moderate and severe PD corresponded to the overall number of PD.

Also, the PISA and the PESA for every tooth in all subjects were estimated in Microsoft Excel spreadsheet. PISA is the area of bleeding of the pocket epithelium (mm^2^) and PESA is the surface area of the root covered with pocket epithelium (mm^2^)^86,87^.

### Covariates

Self-reported sociodemographic characteristics regarding age, race or ethnicity (i.e., Mexican American, Non-Hispanics White, Non-Hispanics Black, other Hispanics and Other race) and highest education level (i.e., less than high school, complete high school or similar, higher than high school). Further, in this analysis we collected data about current smokers (i.e. who had smoked ≥ 100 cigarettes during their lifetime and were still smoking). BMI was calculated as weight in kilograms divided by height in meters squared. Marital status was defined as single, married/living with a partner, divorced/separated/widowed. Diabetes mellitus was categorized as yes or no according to the self-reported questionnaire. Medical conditions were assessed as the sum of binary variables (to note the presence of asthma, congestive heart failure, coronary heart disease, angina, stroke, heart attack, emphysema, overweight, bronchitis, liver, thyroid and cancer) as previously done in Leira et al.^88^.

Blood levels data included WBC count (10^9^/L), monocyte percentage (%), monocyte (10^9^/L), segmented neutrophils percentage (%), segmented neutrophils (10^9^/L), percentage of eosinophils (%), eosinophils (10^9^/L), percentage of basophils (%), basophils (10^9^/L), lymphocyte percentage (%), lymphocyte (10^9^/L), red blood cell (RBC) count (million cells/uL), hemoglobin (g/dL), hematocrit (%), mean cell volume (MCV) (fL), mean cell hemoglobin (MCH) (pg), mean cell hemoglobin concentration (MCHC) (g/dL), percentage of red cell distribution (RCD) width (%), percentage of platelet count (%), mean platelet volume (MPV) (fL), CRP (mg/dL). Overall, the markers expected to present association were WBC, Segmented neutrophils percentage, RBC, Hemoglobin, Hematocrit, MCV, MCH and MCHC according to Botelho^89^.

We used PISA as a proxy for periodontal (local) inflammation and serum levels of CRP as a proxy for systemic inflammation, respectively^88,90^.

### Data management, test methods and analysis

Data from the NHANES 2001–2002 and 2003–2004 were uploaded through SAS Universal Viewer for Windows and transformed to SPSS version 25.0 for Macintosh (Armonk, New York, IBM Corp.). All periodontal database was transferred to a Microsoft Excel with an appropriate algorithm to assess the periodontal case definition. Descriptive measures are described as mean ± SD for continuous variables, and number of cases (n) and percentage (%) for categorical variables. Mean values explicit comparison was performed by t-Student test when data assumptions for the application of this test were met (normality and homoscedasticity). When those assumptions were not verified, Mann–Whitney was used. For categorical variables we compared baseline variables according to BV patients diagnosed using Chi-square test. Additionally, in order to investigate the trend in CRP levels according to PISA values in patients with and without BV, we explore a graph using scatterplots from ggplot2 package for R (version 4.0), and the tendency was computed and fitted via ‘geom_smooth’. A multivariate stepwise adjusted logistic regression method was used to model the influence of the investigated factors towards the presence of PD in patients with BV. Logistic regression analyses were performed, accounting for PD staging, for all women and as a function of positive diagnosis of BV. Logistic regression analyses calculated the OR and the 95% CI, for different adjustment levels. Model variables were selected among clinical and demographic characteristics. Following the initial crude model (model 1), five progressively adjusted models were calculated (model 2: age; model 3: age and smoking habits; model 4: age, smoking habits and BMI; model 5: age, smoking habits, BMI and diabetes mellitus; model 6: age, smoking habits, BMI, diabetes mellitus and number of systemic diseases excluding diabetes mellitus). A significance level of 5% was set in all inferential analyses.

## Supplementary Information

Supplementary information.
